# Efficacious Intermittent Dosing of a Novel JAK2 Inhibitor in Mouse Models of Polycythemia Vera

**DOI:** 10.1371/journal.pone.0037207

**Published:** 2012-05-18

**Authors:** Manfred Kraus, Yuxun Wang, Dan Aleksandrowicz, Eric Bachman, Alexander A. Szewczak, Deborah Walker, Lin Xu, Melaney Bouthillette, Kaleen M. Childers, Brian Dolinski, Andrew M. Haidle, Johnny Kopinja, Linda Lee, Jongwon Lim, Kevin D. Little, Yanhong Ma, Anjili Mathur, Jan-Rung Mo, Erin O’Hare, Ryan D. Otte, Brandon M. Taoka, Wenxian Wang, Hong Yin, Anna A. Zabierek, Weisheng Zhang, Shuxia Zhao, Joe Zhu, Jonathan R. Young, C. Gary Marshall

**Affiliations:** Departments of DMPK, in vitro Sciences, in vivo Sciences, Medicinal Chemistry, Basic Pharmaceutical Sciences and Oncology, Merck Research Laboratories, Boston, Massachusetts, United States of America; Emory University, United States of America

## Abstract

A high percentage of patients with the myeloproliferative disorder polycythemia vera (PV) harbor a Val617→Phe activating mutation in the Janus kinase 2 (JAK2) gene, and both cell culture and mouse models have established a functional role for this mutation in the development of this disease. We describe the properties of MRLB-11055, a highly potent inhibitor of both the WT and V617F forms of JAK2, that has therapeutic efficacy in erythropoietin (EPO)-driven and *JAK2V617F*-driven mouse models of PV. In cultured cells, MRLB-11055 blocked proliferation and induced apoptosis in a manner consistent with JAK2 pathway inhibition. MRLB-11055 effectively prevented EPO-induced STAT5 activation in the peripheral blood of acutely dosed mice, and could prevent EPO-induced splenomegaly and erythrocytosis in chronically dosed mice. In a bone marrow reconstituted *JAK2V617F*-luciferase murine PV model, MRLB-11055 rapidly reduced the burden of *JAK2V617F*-expressing cells from both the spleen and the bone marrow. Using real-time *in vivo* imaging, we examined the kinetics of disease regression and resurgence, enabling the development of an intermittent dosing schedule that achieved significant reductions in both erythroid and myeloid populations with minimal impact on lymphoid cells. Our studies provide a rationale for the use of non-continuous treatment to provide optimal therapy for PV patients.

## Introduction

Polycythemia vera (PV), essential thrombocythaemia (ET) and primary myelofibrosis (PMF) are all conditions that are classified as “Philadelphia-chromosome negative chronic myeloproliferative disorders”. PV is one of the more prevalent, afflicting an estimated 65,000 patients in the United States alone [1], and is associated with splenomegaly, erythrocytosis, thrombocytosis and leukocytosis. Standard of care treatment for PV is phlebotomy supplemented with low-dose aspirin, and for intermediate to high risk patients follow-up hydroxyurea can be prescribed [2], [3]. While these treatment regimens have provided an excellent survival benefit, patients still suffer from reduced quality of life, owing to chronic fatigue, pruritis and bone pain [4]. Additionally patients are at significant risk of transformation to hematological malignancies such as AML.

In 2005, several groups independently discovered a somatic mutation of the gene encoding JAK2 in a high percentage (>95%) of patients with PV, and to a lesser extent, ET and PMF [5]–[9]. A single valine to phenylalanine mutation at position 617, located in a pseudokinase domain thought to negatively regulate the adjacent kinase domain, results in increased JAK2 autophosphorylation, and subsequent activation of downstream signaling networks. Mutation of JAK2 confers cytokine-independent proliferation and survival of a previously EPO-dependent cell line, consistent with its role in mediating erythropoietin (EPO) signaling. Remarkably, reconstitution of irradiated mice with transduced bone marrow expressing *JAK2V617F* leads to a condition that strongly resembles PV within 4–6 weeks, with overt erythrocytosis, splenomegaly, and in some strains of mice, leukocytosis [10]–[12]. Treatment with JAK2 inhibitors can attenuate these symptoms[13]–[15], thus, there is genetic, cell based, and *in vivo* evidence to suggest a functional role for mutant JAK2 in the pathology of PV, and it is reasonable to predict that targeting the JAK2 protein could have therapeutic benefit in this patient population.

In fact, the MPD community has been eagerly anticipating the development of JAK inhibitors, and several are currently being tested in clinical trials [16]–[18]. Given the broad role of JAK kinases in hematopoiesis, a key challenge will be not only the discovery of high quality targeted agents, but also effective methods of their use, as chronic, profound inhibition would likely be problematic. We have recently reported the discovery of a potent, orally active inhibitor of JAK2 [19]. In this study we report the biological characterization of this inhibitor (MRLB-11055) and its use to identify a safe and efficacious dosing schedule in a JAK2^V617F^-dependent model of PV.

## Materials and Methods

### 1. Reagents, Cell Lines, Ethics Statement

MRLB-11055 was synthesized as described previously [20].

Antibodies used were as follows. Phospho-STAT3 (Tyr705) D3A7 rabbit monoclonal antibody, Cell Signaling Technology (CST) #9145. STAT3 rabbit polyclonal antibody, CST #9132. Phospho-Src Family (Tyr416) rabbit polyclonal antibody, CST #2101. Polysorbate 80, Fluka. PEG400, Sigma Aldrich. HPβCD, Cyclodextrin Technologies Development Holdings.

BaF3 cells were obtained from the laboratory of Dr. Gary Gilliland, Brigham & Women’s Hospital. All cell lines were obtained from ATCC. All animal studies were performed according to approved protocols by Merck Boston’s Institutional Animal Care and Use Committee (06-04-019; 07-04-033; 08-04-032).

### 2. *In vitro* Assays

#### 2.1. JAK enzyme assays

JAK1, JAK2, JAK3, and TYK2 kinase activity assays were performed as described previously [21] using HTRF detection technology and the peptide substrate amino hexanoyl biotin-EQEDEPEGDYFEWLE-NH_2_. Each reaction was incubated between 60 and 80 min at room temperature. Final conditions were as follows: JAK1, 400 pM enzyme, Hepes pH 7.5, 10 mM MgCl_2_, 0.01% Brij-35, 1 mM EGTA, 0.1 mg/ml BSA), 2 mM DTT, 2 µM peptide substrate, 25 µM MgATP, 5% DMSO and the desired concentration of subject compound; JAK2, 25 pM enzyme (JH1, JH1–JH2^wt^, JH1–JH2^V617F^ domains), 2 µM peptide substrate, 15 µM MgATP, 5 mM MgCl_2_, 100 mM NaCl, 2 mM DTT, 0.1 mg/ml BSA, 50 mM Tris (pH 7.4), 5% DMSO and the desired concentration of subject compound; JAK3, 250 pM enzyme, 50 mM Hepes pH 7.5, 10 mM MgCl_2_, 0.01% Brij-35, 1 mM EGTA, 0.1 mg/ml BSA), 2 mM DTT, 2.0 µM peptide substrate, 25 µM MgATP, 5% DMSO, and the desired concentration of subject compound; TYK2, 125 pM enzyme, 50 mM Hepes pH 7.5, 10 mM MgCl_2_, 0.01% Brij-35, 1 mM EGTA, 0.1 mg/ml BSA, 2 mM DTT, 2.0 µM peptide substrate, 15 µM MgATP, 5% DMSO and the desired concentration of subject compound.

Kinase profiling was conducted by Millipore (Billerica, MA).

#### 2.2. Cell phosphoprotein assays

Measurement of pSTAT5 in cells was performed essentially as described previously [21] using an AlphaScreen™ SureFire™ (Perkin Elmer) assay. BaF3 (JAK2 WT), BaF3 (JAK2 V617F), and CTLL-2 cells were exposed to compound for 60 min prior to lysis and subsequent detection of pSTAT5 via AlphaScreen technology. Prior to compound treatment, BaF3 (JAK2 WT) and CTLL-2 cells were cytokine starved overnight, with EPO or IL-2 added 15 minutes prior to lysis, respectively.

Phosphorylated and unphosphorylated forms of STAT3 were measured by Western Blot. A549 cells (600,000) were treated with inhibitor for 45 minutes, then stimulated with IL-6 (10 ng/mL) for 15 minutes. Cells were lysed in 30 mM Tris (pH7.5), 5 mM EDTA, 50 mM NaCl, 30 mM NaPPi, 50 mM NaF, 0.5% IGEPAL, 1% Triton, 10% Glycerol, 1 complete Protease Inhibitor Cocktail Tablet, mini (Roche), and 1 PhosSTOP Phosphatase Inhibitor Cocktail Tablet (Roche). Proteins (50 µg) were seperated with a 7.5% SDS-PAGE gel, and transferred onto a nitrocellulose membrane. Proteins were detected using antibodies described in the Materials section, as per the manufacturer protocol.

#### 2.3. Cell phenotypic assays

Cell proliferation was measured by ViaLight (Lonza Group Ltd) assay as follows. Cells (1000–2000) were seeded into 384-well plates and incubated 16 hours @ 37°C, 5% CO_2_. Inhibitors (dissolved in DMSO) were added such that the final concentration of DMSO did not exceed 0.25%, and cells were further incubated for 48–72 hrs. Cells were lysed and ATP detected as per the manufacturer protocol.

Cell survival was measured by ApoDirect (BD Biosciences) assay as follows. BaF3 cells were grown at 37°C, 5% CO_2_, and aliquots (2 × 10^6^) were treated with inhibitor (various concentration dissolved in DMSO) for 24 hours and then fixed and stained as per manufacturer protocol.

#### 2.4 Human Progenitor Colony Formation Assays

Assays were conducted by Stem Cell Technologies Inc. using MethoCult^™^ media (methylcellulose-based media containing cytokines) with or without EPO. MRL-11055 was added to media at the specified concentrations, and cells (from either normal donors or PV patients) were then added at a density optimized for colony formation. Each condition was plated into triplicate plates and incubated at 37°C, 5% CO_2_. Following 14 days in culture, the colonies were assessed and scored based on size and morphology. All samples were obtained anonymously.

### 3. Darbepoetin-Induced Model of PV

#### 3.1. Formulation and Dosing of MRLB-11055

C57BL/6 mice (Charles River Laboratories, Wilmington, MA) aged 4–6 weeks were dosed with MRLB-11055 in vehicle (10%v/v polysorbate 80) or with vehicle alone. All dosing was done by oral gavage (10 ml/kg body weight/day).

#### 3.2. Acute PK/PD

Blood sampling and measurement of pSTAT5 in the blood of darbepoetin-stimulated mice were measured as described previously [22]. The level of MRLB-11055 in the blood of animals was measured by diluting blood samples (10 uL) with citrate buffer (30 uL) and precipitating with acetonitrile (200 uL) containing a structural analog as internal standard. Precipitated samples were filtered, water (200 uL) was added and the sample injected onto a C_18_ column (Waters Atlantis T3, 2.1 mm x 30 mm). Electrospray ionization with multiple reaction monitoring was used for MS/MS detection.

### 3.3. Efficacy Endpoints

Inhibition of darbepoetin-induced polycythemia and splenomegaly by MRLB-11055 was measured as described previously [22].

### 4. Bone Marrow Transplant Mice Expressing JAK2^V617F^-Luciferase

#### 4.1. Viral Production and Bone Marrow Transplantation

Production of retrovirus containing cDNA of JAK2 V617F fused to luciferase 2 was performed as described previously [23] as was transplantation of bone marrow cells infected with virus into irradiated mice.

#### 4.2. Bioluminescence Imaging (BLI)

Imaging of mice expressing JAK2 V617F-Luciferase was conducted as described previously [23].

#### 4.3. JAK2 V617F allele-specific real-time PCR

Measurement of the level of JAK2 V617F allele in the tissues of transplanted mice was done as described previously [23].

### 5 Flow Cytometry

Cells were stained in PBS, 0.5% BSA with antibodies for 15 min on ice. Stained cells were acquired on a 4-laser LSR-II (BD Biosciences) and data analyzed using FlowJo software (Treestar). Dead cells were labeled with the blue fluorescent LIVE/DEAD reactive dye (Invitrogen) and excluded from the analysis.

Monoclonal antibodies (anti-mouse): CD3, CD4, CD5, CD8, CD11b, CD19, CD21/35, CD23, CD25, CD43, CD44, CD49b, CD69, CD71, CD93, CD117, B220, GR-1, IgD, NK1.1, NKG2A/C/E, NKG2D, Ter119 were purchased from BD Biosciences; CD4, CD8, CD49b, CD62L, TCRβ, TCRγδ from eBioscience; GR-1 from Invitrogen; CD43 from Miltenyi Biotec. Goat anti-mouse IgM-FITC, (μ chain specific) from Jackson Immuno Research.

## Results

### *In vitro* Properties of MRLB-11055

**Figure 1:**
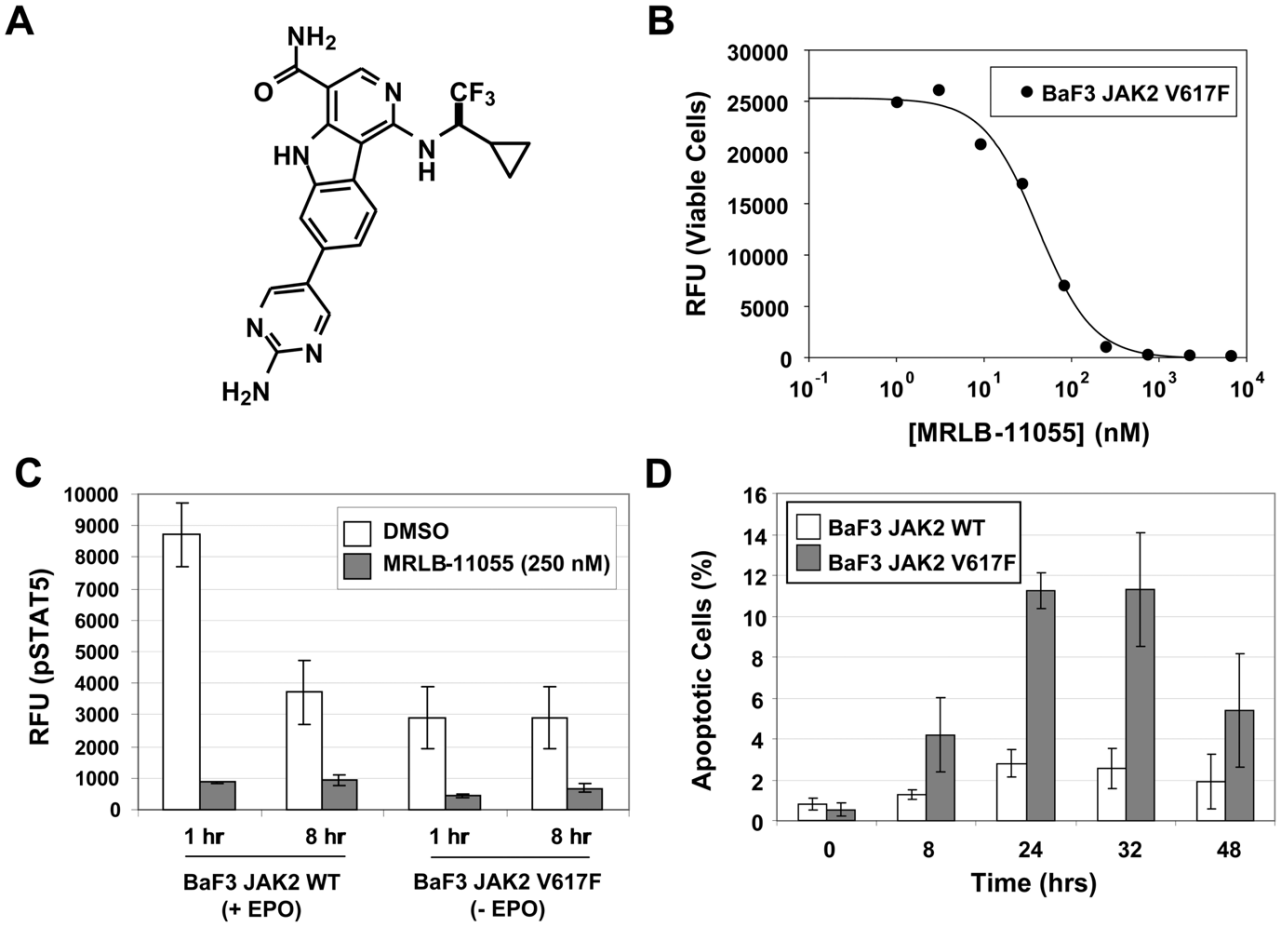
Effect of MRLB-11055 on JAK2-dependent cell lines grown *in vitro*. A. Structure of MRLB-11055. B. Effect on proliferation of BaF3 JAK2 V617F cells over 48 hours. C. Effect on phosphoSTAT5 levels in JAK2-expressing BaF3 cells. D. Effect of 75 nM MRLB-11055 on level of apoptosis in BaF3 JAK2 cells over 48 hours.

MRLB-11055 (Figure 1A) potently inhibits the ability of both wild-type JAK2 (JAK2^WT^) and mutant JAK2 (JAK2^V617F^) to phosphorylate a peptide substrate in cell-free assays (Table 1). MRLB-11055 inhibits the JH1 kinase domain in an ATP-competitive manner (data not shown), and therefore binds to the ATP-binding pocket of JAK2. In the IL-3 dependent BaF3 cell line, expression of both EPO receptor and either WT or V617F JAK2 kinase, causes proliferation to become dependent on EPO (JAK2^WT^ case) or independent of growth factor (JAK2^V617F^ case), respectively [8]. MRLB-11055 potently inhibited proliferation in both of these engineered cell lines (Table 1, Figure 1B), in a manner that correlated closely with inhibition of phosphorylation of the JAK2 substrate, STAT5 (Table 1, Figure 1C). As seen in the cell-free assays, MRLB-11055 had similar potency against both the mutant and WT form of JAK2 in BaF3 cell proliferation assays. This indifference was also observed for inhibition of colonies formed from the blood of PV patients and from healthy donors (Figure S1). In TUNEL assays that measure apoptosis, however, JAK2^V617F^-expressing cell lines demonstrate significantly greater response to MRLB-11055 than their WT counterpart (Figure 1D). Taken together, MRLB-11055 is a potent inhibitor of both JAK2^WT^ and JAK2^V617F^ in both cell-free and cell-based assays, and there is evidence that JAK2^V617F^ cells are more dependent on the activated JAK2 pathway for survival.

Selectivity of MRLB-11055 was assessed against recombinant JAK family members using a variety of methods (Table 1). In cell-free assays, MRLB-11055 demonstrated excellent selectivity for JAK2 over JAK3 (>3000-fold), while selectivity over JAK1 and Tyk2 was more limited (20-fold and 2-fold, respectively). In CTLL-2 cells, IL-2 stimulation leads to phosphorylation of STAT5 via both JAK1 and JAK3. MRLB-11055 inhibited this activity with an IC_50_ of 66 nM, 6-times less potently than the inhibition of JAK2^V617F^-mediated pSTAT5 in BaF3 cells. However, in proliferation assays, MRLB-11055 was actually 2-fold more potent against the IL-2-dependent CTLL cells than the JAK2^V617F^ BaF3 cells, consistent with a greater functional dependence on JAK-STAT signaling in this T-lymphocyte derived cell line. MRLB-11055 was also tested for its ability to inhibit a broad panel of recombinant kinases in cell-free assays. Of the 220 tested kinases, JAK2 was the most potently inhibited, and 198 kinases yielded an IC_50_ value that was greater than 25-fold of the JAK2 IC_50_. Among these off-target kinases were several Src-kinase family members (Table S1). To assess the consequence of the inhibition of these 22 kinases (and potentially other cellular kinases that are not available for cell-free assay), we tested the effect of MRLB-11055 on the proliferation of several lung epithelial cancer cell lines, including H2122 cells (Table 1). On average, the IC_50_ for inhibition of these lines was 50–70-fold higher than for JAK2-dependent cell lines. We conclude that MRLB-11055 has good JAK2 selectivity that specifically inhibits growth of JAK2 dependent cell lines and is suitable to perform therapeutic efficacy experiments *in vivo*.

**Table 1:** *In vitro* Profile of MRLB-11055.

Category	Activity	IC_50_ (nM)^a^
Cell-free JAK2^b^	JAK2 (JH1)^WT^	0.08
	JAK2 (JH1–JH2)^WT^	0.78
	JAK2 (JH1–JH2)^V617F^	0.35
		
Cell pSTAT5^c^(1 hour)	BaF3^d^ (JAK2 WT)	23
	BaF3 (JAK2 V617F)	11
	CTLL-2^e^	66
		
Cell Proliferation^f^(48 hour)	BaF3^d^ (JAK2 WT)	14
	BaF3 (JAK2 V617F)	29
	CTLL-2^f^	13
	H2122^g^	1900
		
Cell Apoptosis^h^(24 hour)	BaF3^d^ (JAK2 WT)	260
	BaF3 (JAK2 V617F)	135
		
Cell-free Kinase Selectivity	TYK2 (JH1)	0.18
	JAK1 (JH1)	1.5
	JAK3 (JH1)	300
	198/220 kinases^i^	>25-fold over JAK2

a) Mean value, n = 3.

b) Kinase activity, HTRF assay.

c) pJAK2 and pSTAT5 activity measured via AlphaScreen and BeadLyte assays.

d) EPO-stimulated.

e) IL2-stimulated CTLL-2 cells, JAK1 and JAK3 dependent.

f) Cell growth, Vialight assay.

g) Epithelial lung cancer cell line.

h) Apoptosis determined by ApoDirect measurement of fragmented DNA. Values shown are EC_50._

i) See Table S1 for all kinases within 25-fold. Fold selectivity based on JAK2 (JH1-JH2) IC_50._

### Effect of MRLB-11055 on JAK2 Pathway Activity *in vivo*

**Figure 2:**
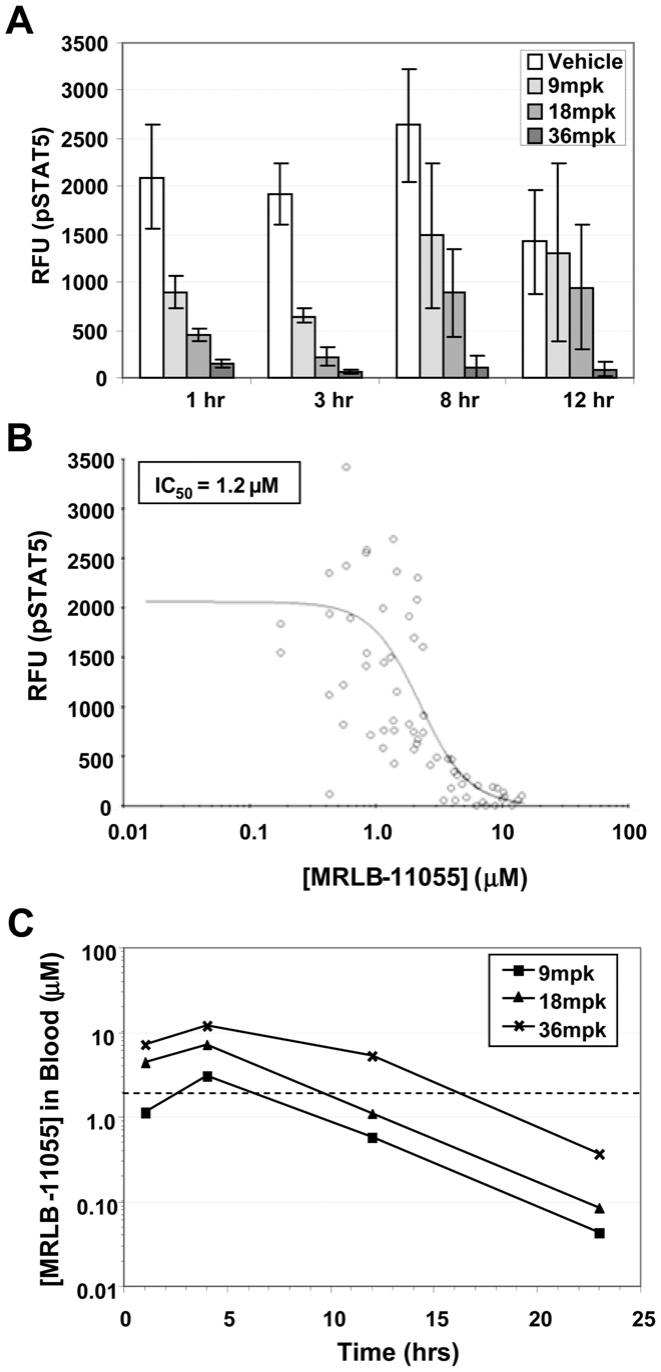
Exposure and target engagement of MRLB-11055 in the peripheral blood of C57BL/6 mice stimulated with darbepoetin. A. Effect of MRLB-11055 on phosphoSTAT5 levels at various times post-dose. B. Calculation of IC50 value for inhibition of phosphoSTAT5. C. PK of 3 doses of MRLB-11055 in mouse blood, with calculated IC50 superimposed (dashed line).

We have previously established that C57BL/6 mice injected with the EPO analog darbepoetin display elevated levels of pSTAT5 in the peripheral blood [22]. To assess the ability of MRLB-11055 to inhibit EPO signal transduction pathways *in vivo*, we orally administered compound to C57BL/6 mice that had been simultaneously injected with darbepoetin and measured pSTAT5 levels in blood over time. As shown in Figure 2A, pSTAT5 was reduced in a dose-proportional manner, and >90% inhibition was maintained over 12 hours at a dose of 36 mg/kg (mpk). The IC_50_ for MRLB-11055 in this system was determined to be 1.2 µM (Figure 2B). MRLB-11055 demonstrated dose-proportional exposure in the peripheral blood of mice, and at 36 mpk we predict that MRLB-11055 remains above the pSTAT5 IC_50_ for approximately 16 hours (Figure 2C).

### Effect of MRLB-11055 on Darbepoetin-Induced Model of Polycythemia

**Figure 3:**
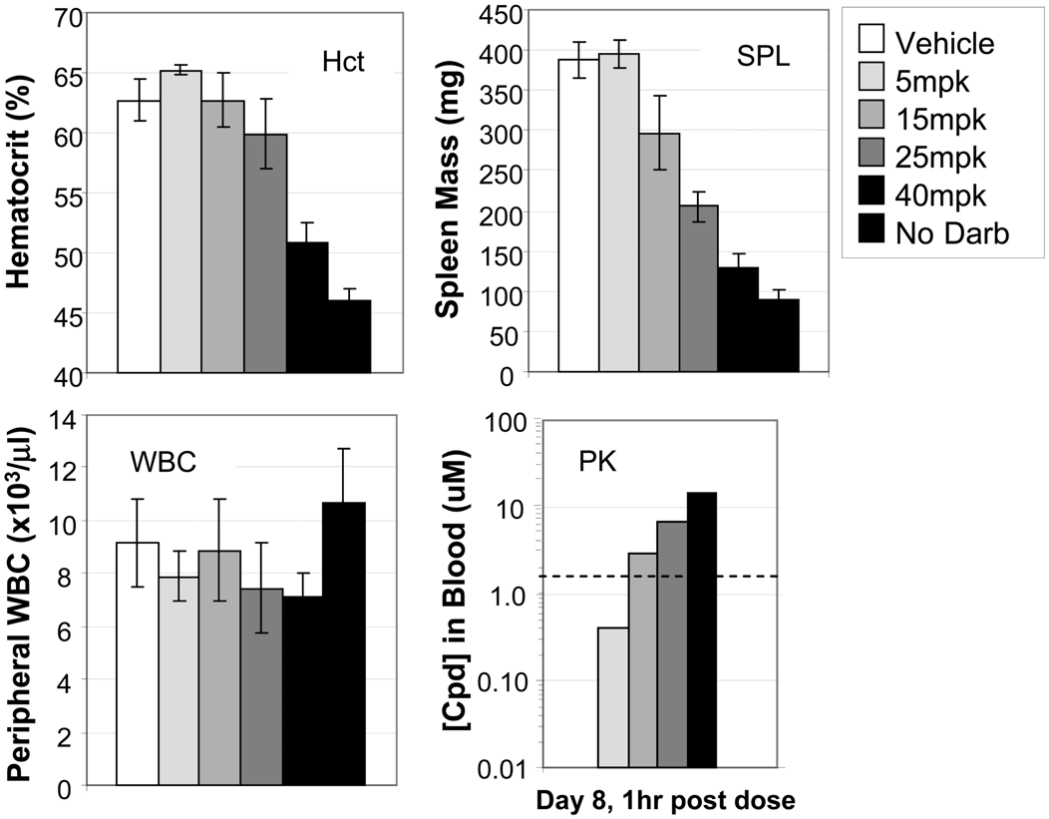
Effect of MRLB-11055 in a Darbepoetin-Induced PV Efficacy Model. The ability of MRLB-11055 to prevent darbepoetin-induced increases in hematocrit (Hct) and spleen mass (SPL) over 7 days is shown, as is the impact of MRLB-11055 on white blood cells (WBC) and its concentration in blood (PK). Dashed line indicates *in vivo* IC50 value.

Mice repeatedly administered the EPO analog darbepoetin develop erythrocytosis and splenomegaly – both major diagnostic criteria for PV - over a period of 7 days [22]. The effectiveness of MRLB-11055 in preventing the development of the polycythemic phenotype was assessed by orally administering drug once daily at doses of 5, 15, 25 and 40 mpk and comparing disease endpoints to that of mice given only vehicle and mice not given any darbepoetin (Figure 3). Elevated hematocrit (Hct) and spleen weight (SPL) were prevented in a dose-dependent manner, with the highest dose achieving efficacy levels of 76% and 87%, respectively. MRLB-11055 demonstrated dose dependent exposure in the blood that correlated with its effect on the polycythemic phenotype. At the highest dose of 40 mpk, MRLB-11055 achieved a concentration equal to about 10 times its *in vivo* pSTAT5 IC_50_ value, when measured one hour after administration on the last day of the experiment. MRLB-11055 demonstrated a dose-dependent trend towards WBC reduction, that did not reach statistical significance. Thus, MRLB-11055 was effective at preventing acute development of a PV-like disease driven by *wild-type* JAK2.

### Effect of MRLB-11055 on JAK2^V617F^-Luciferase Expressing Cells in Bone Marrow Transplant Mice

**Figure 4:**
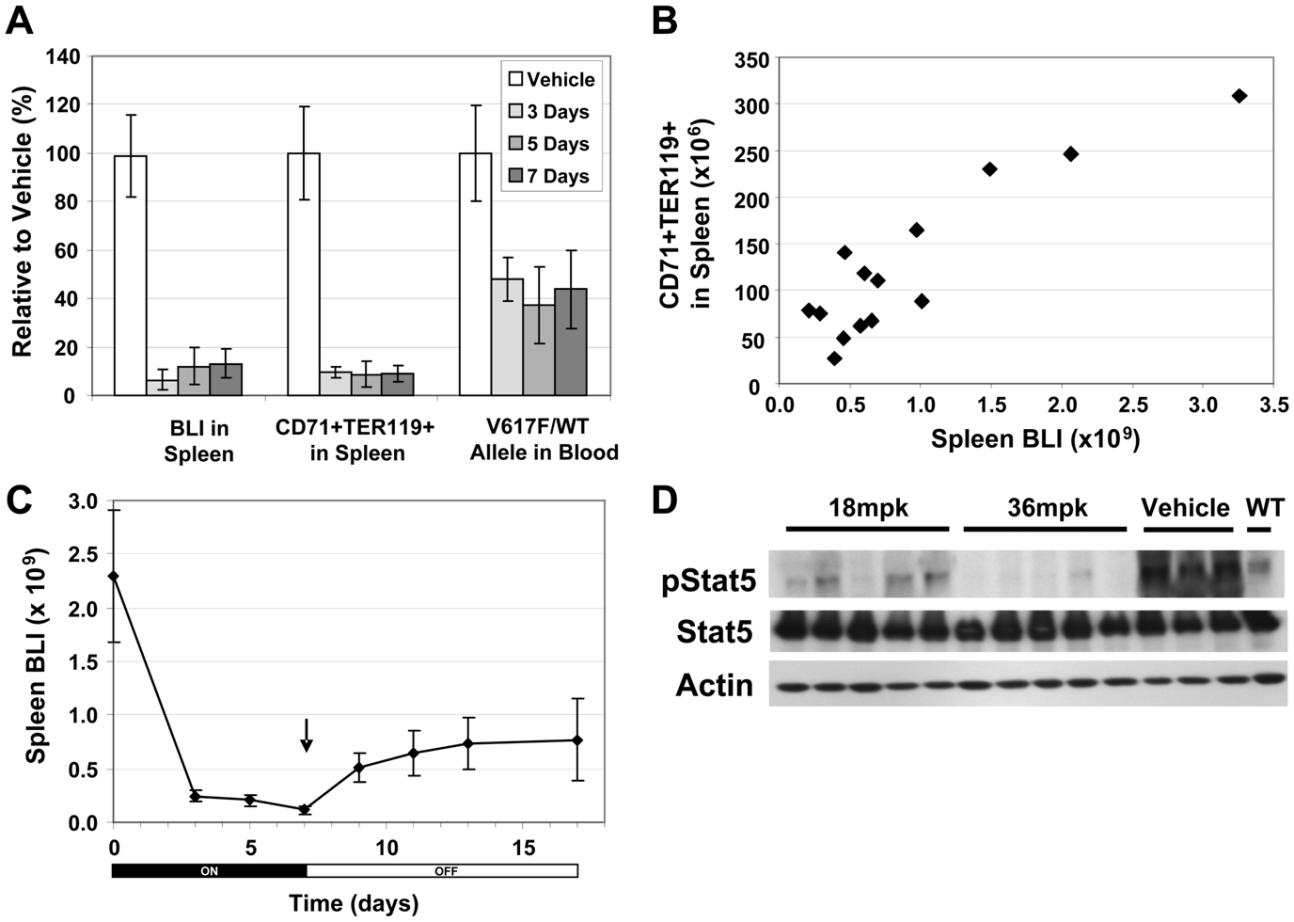
Effect of MRLB-11055 on Target Tissue in JAK2 V617F-Luc2 Mice. A. Time dependence of the effect of 54 mpk MRLB-11055 on key V617F-dependent endpoints. B. Correlation between BLI and CD71+TER119+ in spleen on Day 14 C. BLI recovery after treatment cessation on Day 7. D. Effect of MRLB-11055 on pSTAT5 in the spleen of V617F mice.

We have previously described a model of PV in which lethally irradiated mice receive bone marrow that co-expresses JAK2^V617F^ with luciferase [23]. These mice develop a robust PV phenotype 4 weeks after transplantation and cells expressing the transduced genes, which can be monitored in real-time with bioluminescent imaging methodology, are observed to expand rapidly in hematopoetic compartments, particularly the spleen. To determine the effect of MRLB-11055 on JAK2^V617F^-expressing cells in this model system, we orally administered drug once daily at a dose of 54 mpk for 3, 5 and 7 days, and examined bioluminescent intensity (BLI) and erythroid progenitor cells, identified as CD71^+^TER119^+^ in spleen, as well as V617F allele burden (measured by qPCR) in peripheral blood (Figure 4A). Significant reductions in all three endpoints were observed at the earliest timepoint of Day 3, with no further benefit from additional days of treatment out to Day 7. Similar reductions in BLI were observed in bone marrow and in lateral side of the mouse, including spleen (data not shown). We also examined the correlation between BLI and CD71^+^TER119^+^ cells in individual mice treated with MRLB-11055. Figure 4B demonstrates a good correlation between these two endpoints, suggesting that BLI (repeatedly available from a given mouse) is a good methodology for repeated measurements and surrogate for splenic erythoid progenitor fraction size (whose measurement requires sacrifice of the mouse). We further measured the effect of cessation of MRLB-11055 treatment on spleen BLI in mice treated once daily for 7 days. As shown in Figure 4C, some recovery of JAK2^V617F^-expressing cells was observed, however the recovery was slow and remained below pre-treatment levels. As shown in Figure 4D, the level of pSTAT5 in spleens of JAK2^V617F^-Luciferase mice was significantly inhibited by MRLB-11055 (1 hour post-dose), consistent with the observed effects on BLI, erythroid progenitors and JAK2^V617F^. These experiments collectively demonstrate that MRLB-11055 is effective at treating early efficacy endpoints in a JAK2^V617F^ -driven model of PV.

### Effect of Multi-cycle Intermittent Dosing of MRLB-11055 on PV Phenotype in BMT Mice Expressing JAK2^V617F^-Luciferase

**Figure 5:**
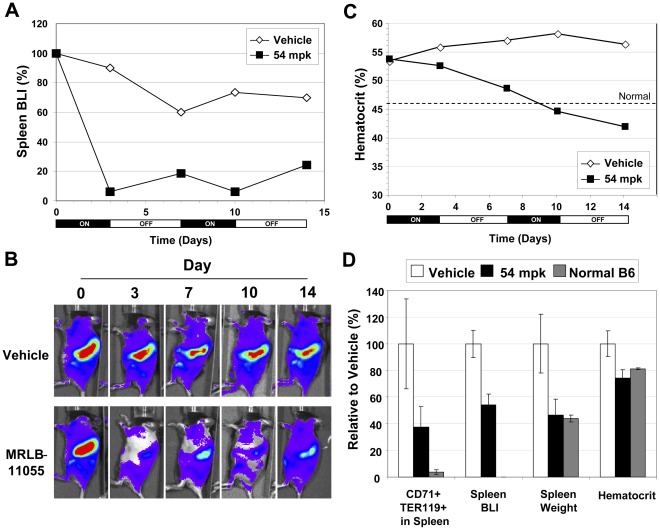
Effect of 2 Cycles of Intermittent Dosing (3 days on, 4 days off) of MRLB-11055 on V617F-Luc2 Mice (N = 10). Effect on A & B. Bioluminescence in spleen C. Hematocrit D. Multiple endpoints at end of study (Day 14).

Having established that 3 days of once daily treatment with MRLB-11055 significantly impacts JAK2^V617F^-expressing cells, with partial resurgence of these cells upon termination of treatment, we next examined the effect of multiple cycles of intermittent dosing of this inhibitor on the PV phenotype in JAK2^V617F^-Luciferase BMT mice. Each cycle consisted of oral administration of 54 mpk once daily for 3 days, followed by a 4 day holiday. Mice were treated with 2 cycles of this regimen upon completing their 3^rd^ week post-transplant, at a time when BLI and CD71^+^TER119^+^ cells in the spleen had reached a plateau, and mice were mildly polycythemic, but hematocrit was still in the process of increasing. When treated in this way, the spleens of mice demonstrated a significant reduction (∼20-fold) in BLI over the first 3 days of treatment. BLI subsequently increased nearly 4-fold over the next 4 days, but it remained about 5-fold less than pre-treatment levels (Figure 5A). The second cycle of treatment and holiday resulted in levels that were similar to those obtained with the first cycle. In comparison, the BLI levels in the spleen of vehicle treated mice decreased slightly over these two cycles (Figure 5B). Hematocrit levels in vehicle treated mice increased slowly over the 2 cycle treatment, from 53% to 56% (Figure 5C). In mice treated with MRLB-11055, however, hematocrit decreased from 53% to 42%. Reductions in hematocrit occurred during both administration and holiday phases of the cycle. At the end of the second holiday period, mice demonstrated a significant reduction in CD71^+^TER119^+^ cells in spleen relative to vehicle controls, as well as normalization in spleen weight (Figure 5D). Similar results were obtained when mice were treated for 4-cycles (Figure S2), demonstrating that intermittent dosing can be efficacious over prolonged periods of time. Thus, MRLB-11055 effectively impacted clinically relevant endpoints in a JAK2^V617F^-dependent model of PV.

### Effect of MRLB-11055 on Major Lymphoid Populations in WT Mice

JAK2 and its family members JAK1 and JAK3 are known to signal downstream of cytokines that play essential roles in the development of lymphoid cell populations [24]. To examine more thoroughly the effects of JAK2 inhibition on lymphoid cells, we analyzed wild-type (C57BL/6) mice treated with 54 mpk MRLB-11055 for 3 days, 6 days or up to 5 cycles of 3 days (“on”) followed by a 4 day holiday (“off”).

**Figure 6:**
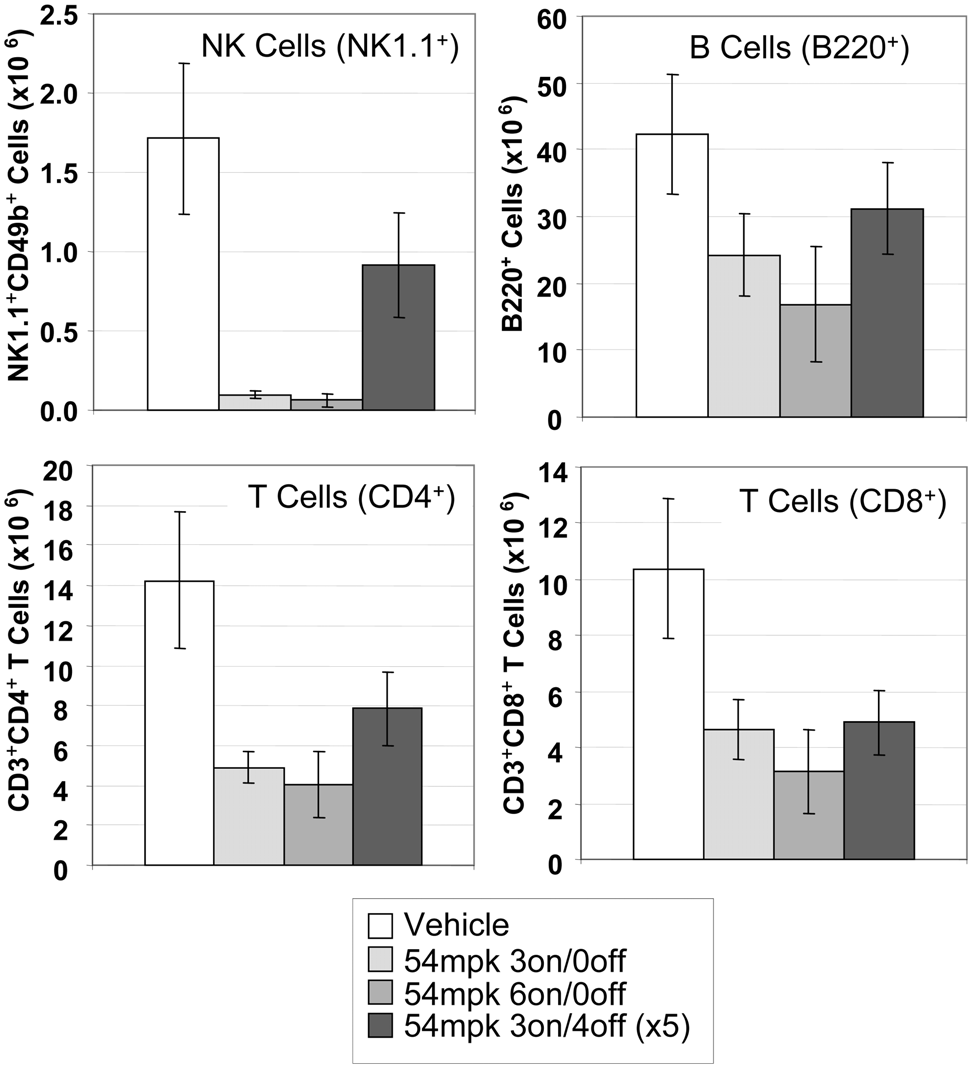
Effect of MRLB-11055 on major lymphoid populations in spleen of WT B6 mice. MRLB-11055 was given for either 3 or 6 days (on), followed up by either a 0 or 4 day holiday (off), for up to 4 cycles. Effects on NK, B and T cells were measured by flow cytometry.

Flow cytometric analysis of splenic populations such as NK cells (NK1.1^+^CD49b^+^), CD4^+^ T-cells (CD3^+^CD4^+^), CD8^+^ T-cells (CD3^+^CD8^+^) and B-cells (B220^+^) revealed a time-dependent decrease of B and T lymphocytes, as well as NK cells, ranging from 2-fold (B220^+^) to 25-fold (NK1.1^+^ cells) over 6 days of continuous treatment with the inhibitor (Figure 6 and Table S2). Intermittent dosing however, when administered for 5 cycles of 3 days on and 4 days off, significantly muted the reductions, not exceeding 2-fold for any of the lymphocytes tested.

Analysis of peripheral blood showed that long-term intermittent inhibition of JAK2 resulted in a lower hematocrit in C57BL/6 mice (Table S3). Consistent with this observation, red blood cell counts and hemoglobin concentrations were lower in the treated animals. The CD71^+^Ter119^+^ erythroid progenitor population in spleen and the reticulocyte count in blood was effectively reduced during MRLB-11055 treatment and recovered quickly during the off-phase, likely in response to the lowered HCT values in treated animals (Tables S2 and S3). JAK2 inhibition did not alter the number of neutrophils but interestingly resulted in an increase of blood platelets.

Similar and consistent results were obtained when analyzing lymphocyte development and erythroid progenitor populations in bone marrow and thymus (Tables S4, S5). These data demonstrate that intermittent dosing of MRLB-11055, at a dose level that was effective at reducing hematocrit and normalizing spleen weight in JAK2^V617F^ mice, was sparing of lymphocyte populations in normal C57BL/6 mice.

## Discussion

Polycythemia vera (PV) is a disease involving biology for which there is a rich history of study. The discovery of the JAK^V617F^ mutation shed light on the mechanism of disease origin and development. However, from the published literature it appears that while JAK2 certainly plays an important role, other elements also likely contribute to the pathological evolution of PV [7], [25]. What is not known is whether inhibition of the constitutively activated JAK2 mutant, signaling aberrantly downstream of the EPO receptor in erythroid and myeloid progenitor cells, will provide an effective improvement in the treatment of PV patients. To that end, several groups have developed pre-clinical models of PV disease [10]–[12], [26], enabling the development of JAK2 inhibitors for evaluation in the clinic. A major challenge in development of any JAK2 inhibitor that is not selective for the V617F form of the enzyme is the expected mechanism-based toxicity, as JAK2 signaling is essential for many biological processes, within the hematopoeitic compartment and beyond [27], [28]. Chronic, high-level inhibition of JAK2 would almost certainly be intolerable, even if only considering the intended target tissues of the erythroid and myeloid lineage. Thus the dosing schedule of a JAK2 inhibitor is likely to be an important consideration in addition to the intrinsic properties of that inhibitor when considering its potential for successful clinical application.

MRLB-11055 is a potent inhibitor of JAK2, however, similar to other described inhibitors of JAK2, it is not selective for JAK2^V617F^ over JAK2^WT^. Despite this lack of selectivity at the enzyme level, cells that are dependent on JAK2^V617F^ for growth are much more likely to commit to apoptosis in the presence of MRLB-11055 than their WT counterparts. This suggests a potential problem of adverse effects arising from chronic systemic JAK2 inhibition, and set the stage for exploring intermittent dosing *in vivo*. The pharmacokinetics of MRLB-11055 in mice was such that we were able to achieve sustained target inhibition each dosing cycle with once daily dosing, enabling efficacy studies. As MRLB-11055 was potent against JAK2^WT^, we were able to demonstrate efficacy in a model where PV-like symptoms, such as erythrocytosis and splenomegaly, could be rapidly generated by treatment of normal C57BL/6 mice with darbepoetin. While an important proof-of-concept for the inhibitor, this model system is preventative, and thus did not allow the interrogation of dosing scheme in the context of an established disease state.

Several mouse models of PV have been described that employ bone marrow transplantation of JAK2^V617F^ to generate a phenotype that bears many of the hallmarks of disease. In all of these models, there is not only an expansion of erythrocytes, but also an expansion of the erythroid progenitor cells, which are their EPO receptor-expressing predecessors. PV patients are known to suffer from an increase in these cells, which appear as endogenous erythroid colonies (eEECs) in *ex-vivo* soft agar assays. In order to evaluate not only the effectiveness of an inhibitor but the optimal dose and schedule of that inhibitor, we reasoned that this progenitor population was the most likely candidate for the direct target tissue for the drug, and hence a key readout. Erythrocytes, as descendants of these cells, are indirectly targeted and with an inherent latency due to their lengthy half-life. Monitoring erythroid progenitors, however, is not readily achieved, and presents a challenge for assessment of optimal treatment time and holiday when developing a dosing schedule. For these reasons, we developed a JAK2^V617F^-Luciferase model system that allows real-time imaging of mutant expressing cell populations, which includes the erythroid progenitor population. A key consideration in the use of this model was the most appropriate stage in the progression of disease for introduction of the JAK2 inhibitor. We chose to administer the inhibitor to the mice at end of the 3rd week post-BMT, at which time they were mildly polycythemic, with hematocrit levels actively rising. This state most closely models the clinical condition of PV patients, who are not allowed to achieve plateau levels of Hct in normal care, and exist in a state of rising hematocrit between phlebotomy treatments.

Under these conditions MRLB-11055 was observed to dramatically reduce the level of both erythroid progenitor cells and BLI in the spleen within a 3 day treatment period. While the exact mechanism of this reduction is not known, it is consistent with the rapid and robust induction of apoptosis in BaF3 cells dependent on JAK2^V617F^ *in vitro*. When MRLB-11055 was removed, V617F-expressing cells immediately began to re-expand, consistent with the previous observation that the JAK2 mutation penetrates into the hematopoeitic stem cell population in these mice [23]. Based on these observed kinetics of reduction and re-expansion, we were able to devise a multi-cycle intermittent dosing scheme aimed at normalizing progenitor populations. Application of this scheme not only prevented further rise of hematocrit in these mice, but actually decreased hematocrit to a level below the normal range. These decreases occurred even during the drug holiday period, clearly demonstrating that JAK inhibition need not be continuous to result in significant efficacy, and that hematocrit levels can be effectively managed by dosing schemes aimed at normalizing erythroid progenitor populations.

JAK inhibitors have been described to have potent effects on lymphocyte subpopulations [29], [30], prompting us to examine these lineages more closely. MRLB-11055 did indeed reduce T, B and NK cell fractions in the spleens of normal C57BL/6 mice when administered continuously at high doses. However, these reductions were significantly alleviated when MRLB-11055 was given intermittently according to the efficacious dosing schedule in the JAK2^V617F^-Luciferase mouse model. As immune function depends on the presence of these lymphoid cells, this data suggests that intermittent dosing could minimize immunodeficiencies induced by treatment with a JAK2 inhibitor.

We recognized that MRLB-11055 had modest selectivity for signaling induced by EPO/JAK2 over signaling induced by IL-2/JAK1/JAK3, a pathway known to play a role in lymphocyte development. Furthermore, MRLB-11055 had little to no selectivity for JAK2 over Src-family kinases and Flt-3 (Table S1), which are also key mediators in the maturation of lymphocytes. To address this, we evaluated the effect of a structurally distinct JAK2 inhibitor with enhanced selectivity over these other signaling molecules. At exposures that resulted in comparable efficacy to MRLB-11055, this inhibitor demonstrated identical reductions in lymphocyte populations (unpublished data). One explanation for these findings is that the reduction in these cell populations is due, at least in part, to inhibition of JAK2 itself, which is consistent with a role of JAK2-dependent cytokines such as IL-12 in lymphocyte development. We have demonstrated that intermittent dosing can attenuate many of the undesirable effects that will likely be associated with the use of JAK2 inhibitors in the treatment of MPD. In addition to signaling downstream of the EPO receptor, JAK2 plays a role in mediating signaling from a variety of molecules, including IFNγ, IL-6, TPO, GM-CSF, prolactin, growth hormone, and angiotensin 1.

The JAK2 inhibitor TG101348 has been described as a molecule that is both efficacious in a murine model of PV and sparing of T lymphocytes [14]. While inhibition of pSTAT5 was clearly demonstrated 2 hours after TG101348 administration, it is not clear how prolonged target inhibition was during dosing. As TG101348 required 42 days of continuous treatment to achieve hematocrit reductions of 18%, it is reasonable to presume that target engagement may have been lower relative to MRLB-11055 for a given dosing cycle. Thus the apparently unperturbed T lymphocyte populations may be explained by a lower level of target engagement. The effect on NK cells, which responded most sensitively to MRLB-11055 inhibition, was not measured with TG101348.

We have demonstrated that intermittent dosing of a JAK2 inhibitor can effectively normalize erythroid progenitor populations and thereby effectively treat conditions of polycythemia and splenomegaly in mouse models of PV. Our data can provide signficant guidance to the clinical development of JAK2 inhibitors. While the kinetics of erythropoesis are likely different in human disease, our data provide proof-of-concept for the use of erythroid progenitor populations as early biomarkers of target tissue efficacy, that could guide development of optimized intermittent dosing schemes to provide patients with improved therapy. Furthermore, our data show that lymphoid populations, in particular NK cells, serve as sensitive biomarkers for JAK inhibitor toxicity that is potentially mechanism-based.

## Supporting Information

Figure S1 **Effect of MRLB-11055 on Human Progenitor Cell Growth Ex-vivo.** A. Effect of dose on colony number. B. Summary of IC50 values across 3 patient samples. C. Representative micrograph of effect on colony growth.
(TIF)

Figure S2 **Effect of 4 Cycles of Intermittent Dosing (3 days on, 4 days off) of MRLB-11055 on V617F-Luc2 Mice (N = 10).** Effect on A & B. Bioluminescence in spleen C. Hematocrit D. Multiple endpoints at end of study (Day 28).
(TIF)

Table S1 **Recombinant Kinase Selectivity Profile of MRLB-11055.** IC50s measured at ATP Km, and fold increase relative to JAK2 is reported.
(DOC)

Table S2 **Effect of MRLB-11055 on Cell Populations in Spleen of Normal Mice. Cell counts measured by Advia.** Cycles refer to 3 days of treatment followed by a 4 day holiday. *p<0.05 in Student T test when comparing vehicle 5 cycle with treatment 5 cycle and vehicle 2 cycle with treatment 2 cycle, resp.
(DOC)

Table S3 **Effect of MRLB-11055 on Cell Populations in Peripheral Blood of Normal Mice.** Cell counts measured by Advia. Cycles refer to 3 days of treatment followed by a 4 day holiday. *p<0.01 and **p<0.001 in Student T test when comparing vehicle with treatment. HCT, hematocrit; RBC, red blood cells; HGB, hemoglobin; WBC, white blood cells.
(DOC)

Table S4 **Effect of MRLB-11055 on Cell Populations in Bone Marrow of Normal Mice.** Cell counts measured by Advia. Cycles refer to 3 days of treatment followed by a 4 day holiday. *p<0.05 in Student T test when comparing vehicle 5 cycle with treatment 5 cycle and vehicle 2 cycle with treatment 2 cycle, resp.
(DOC)

Table S5 **Effect of MRLB-11055 on Cell Populations in Thymus of Normal Mice.** Cell counts measured by Advia. Cycles refer to 3 days of treatment followed by a 4 day holiday. *p<0.05 in Student T test when comparing vehicle 5 cycle with treatment 5 cycle and vehicle 2 cycle with treatment 2 cycle, resp.
(DOC)

## References

[1] Ma X, Vanasse G, Cartmel B, Wang Y, Selinger HA (2008). Prevalence of polycythemia vera and essential thrombocythemia.. Am J Hematol.

[2] Penninga EI, Bjerrum OW (2006). Polycythaemia vera and essential thrombocythaemia: current treatment strategies.. Drugs.

[3] Rice L, Baker KR (2006). Current management of the myeloproliferative disorders: a case-based review.. Arch Pathol Lab Med.

[4] Mesa RA, Niblack J, Wadleigh M, Verstovsek S, Camoriano J (2007). The burden of fatigue and quality of life in myeloproliferative disorders (MPDs): an international Internet-based survey of 1179 MPD patients.. Cancer.

[5] Baxter EJ, Scott LM, Campbell PJ, East C, Fourouclas N (2005). Acquired mutation of the tyrosine kinase JAK2 in human myeloproliferative disorders.. Lancet.

[6] James C, Ugo V, Le Couedic JP, Staerk J, Delhommeau F (2005). A unique clonal JAK2 mutation leading to constitutive signalling causes polycythaemia vera.. Nature.

[7] Kralovics R, Passamonti F, Buser AS, Teo SS, Tiedt R (2005). A gain-of-function mutation of JAK2 in myeloproliferative disorders.. N Engl J Med.

[8] Levine RL, Wadleigh M, Cools J, Ebert BL, Wernig G (2005). Activating mutation in the tyrosine kinase JAK2 in polycythemia vera, essential thrombocythemia, and myeloid metaplasia with myelofibrosis.. Cancer Cell.

[9] Zhao R, Xing S, Li Z, Fu X, Li Q (2005). Identification of an acquired JAK2 mutation in polycythemia vera.. J Biol Chem.

[10] Bumm TG, Elsea C, Corbin AS, Loriaux M, Sherbenou D (2006). Characterization of murine JAK2V617F-positive myeloproliferative disease.. Cancer Res.

[11] Lacout C, Pisani DF, Tulliez M, Gachelin FM, Vainchenker W (2006). JAK2V617F expression in murine hematopoietic cells leads to MPD mimicking human PV with secondary myelofibrosis.. Blood.

[12] Wernig G, Mercher T, Okabe R, Levine RL, Lee BH (2006). Expression of Jak2V617F causes a polycythemia vera-like disease with associated myelofibrosis in a murine bone marrow transplant model.. Blood.

[13] Baffert F, Regnier CH, De Pover A, Pissot-Soldermann C, Tavares GA (2010). Potent and Selective Inhibition of Polycythemia by the Quinoxaline JAK2 Inhibitor NVP-BSK805.. Molecular Cancer Therapeutics.

[14] Wernig G, Kharas MG, Okabe R, Moore SA, Leeman DS (2008). Efficacy of TG101348, a selective JAK2 inhibitor, in treatment of a murine model of JAK2V617F-induced polycythemia vera.. Cancer Cell.

[15] Zaleskas VM, Krause DS, Lazarides K, Patel N, Hu YG (2006). Molecular Pathogenesis and Therapy of Polycythemia Induced in Mice by JAK2 V617F.. Plos One.

[16] Paquette R, Sokol L, Shah NP, Silver RT, List AF (2008). A Phase I study of XL019, a selective JAK2 inhibitor, in patients with polycythemia vera.. ASH Annual Meeting Abstracts.

[17] Pardanani AD, Gotlib J, Jamieson C, Cortes J, Talpaz M (2008). A Phase I study of TG101348, an orally bioavailable JAK2-selective inhibitor, in patients with myelofibrosis.. ASH Annual Meeting Abstracts.

[18] Verstovsek S, Kantarjian HM, Pardanani AD, Thomas D, Cortes J (2008). The JAK inhibitor, INCB018424, demonstrates durable and marked clinical responses in primary myelofibrosis (PMF) and post-polycythemia/essential thrombocythemia myelofibrosis (post PV/ETMF).. ASH Annual Meeting Abstracts.

[19] Lim J, Taoka B, Otte RD, Spencer K, Dinsmore CJ (2011). Discovery of 1-Amino-5*H*-pyrido[4,3-*b*]indol-4-carboxamide Inhibitors of JAK2 for the Treatment of Myeloproliferative Disorders. J Med Chem.. 54,.

[20] Young JR, Lim J, Machacek RM, Taoka MB, Otte DR, inventors; 2009) Preparation of 5H-pyrido[4, 3-b]indole derivatives as janus kinases inhibitorsUnitedStatespatentapplicationWO200907 (5830).

[21] Siu T, Young J, Altman M, Northrup A, Katcher M (2009). Preparation of 7,8-fused-2,6-naphthyridin-1(2H)-ones as inhibitors of janus kinases.. United States patent application WO.

[22] Mathur A, Mo JR, Kraus M, O’Hare E, Sinclair P (2009). An inhibitor of Janus kinase 2 prevents polycythemia in mice.. Biochem Pharmacol.

[23] Ma Y, Zhao S, Zhu J, Bettano KA, Qu X (2009). Real-time bioluminescence imaging of polycythemia vera development in mice.. Biochim Biophys Acta.

[24] Ghoreschi K, Laurence A, O’Shea JJ (2009). Janus kinases in immune cell signaling.. Immunol Rev.

[25] Campbell PJ, Baxter EJ, Beer PA, Scott LM, Bench AJ (2006). Mutation of JAK2 in the myeloproliferative disorders: timing, clonality studies, cytogenetic associations, and role in leukemic transformation.. Blood.

[26] Mullally A, Lane SW, Ball B, Megerdichian C, Okabe R (2010). Physiological Jak2V617F expression causes a lethal myeloproliferative neoplasm with differential effects on hematopoietic stem and progenitor cells.. Cancer Cell.

[27] Marrero MB (2005). Introduction to JAK/STAT signaling and the vasculature.. Vascul Pharmacol.

[28] Parganas E, Wang D, Stravopodis D, Topham DJ, Marine JC (1998). Jak2 is essential for signaling through a variety of cytokine receptors.. Cell.

[29] Changelian PS, Flanagan ME, Ball DJ, Kent CR, Magnuson KS (2003). Prevention of organ allograft rejection by a specific Janus kinase 3 inhibitor.. Science.

[30] Conklyn M, Andresen C, Changelian P, Kudlacz E (2004). The JAK3 inhibitor CP-690550 selectively reduces NK and CD8+ cell numbers in cynomolgus monkey blood following chronic oral dosing.. J Leukoc Biol.

