# Supplementary figures and images for: Efficacious Intermittent Dosing of a Novel JAK2 Inhibitor in Mouse Models of Polycythemia Vera
Source: PLoS One. 2012 May 18;7(5):e37207. doi: 10.1371/journal.pone.0037207 (PMC3356383; doi:10.1371/journal.pone.0037207)

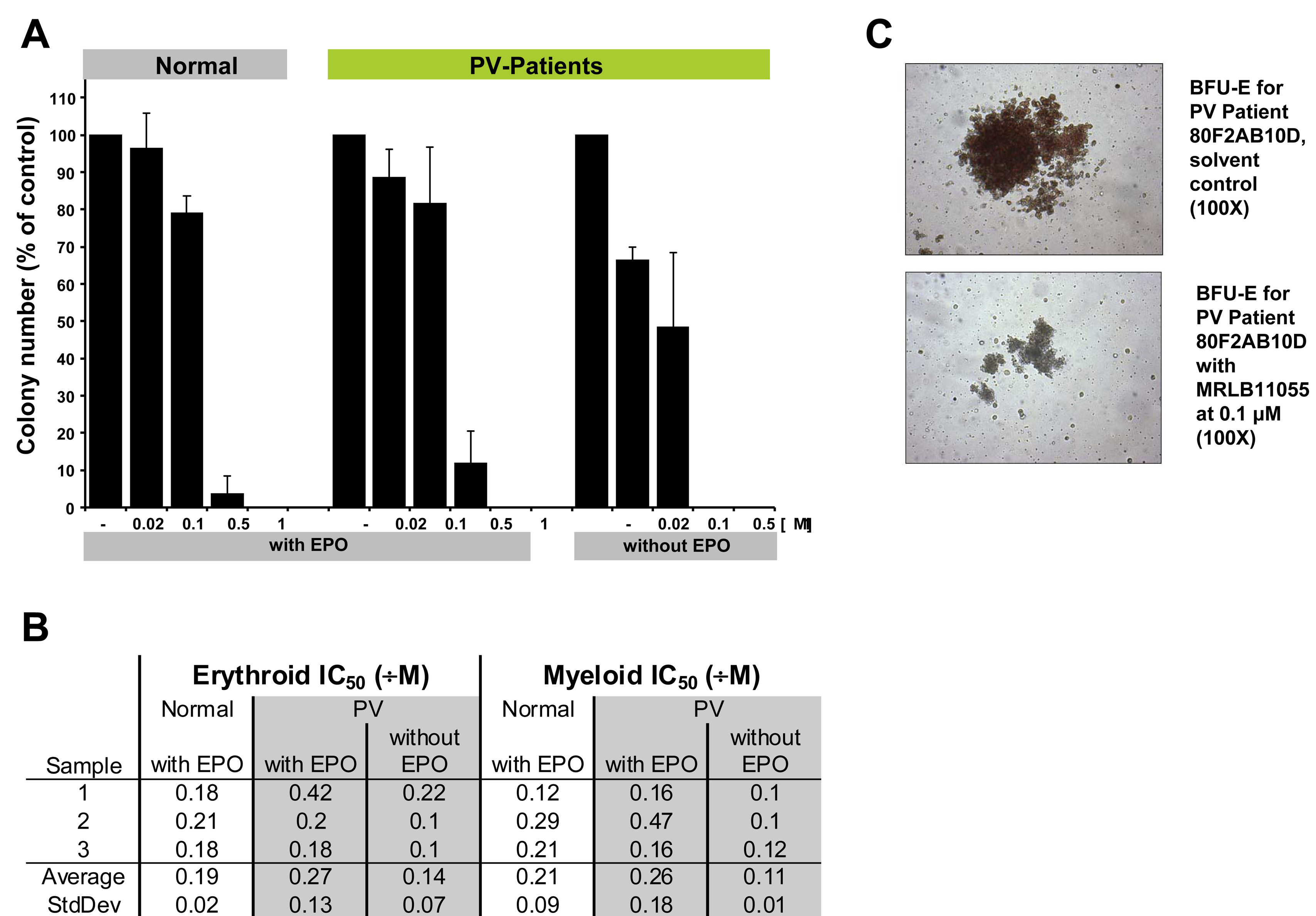

Supplement: Figure S1 — Effect of MRLB-11055 on Human Progenitor Cell Growth Ex-vivo. A. Effect of dose on colony number. B. Summary of IC50 values across 3 patient samples. C. Representative micrograph of effect on colony growth. (TIF) [file pone.0037207.s001.tif]

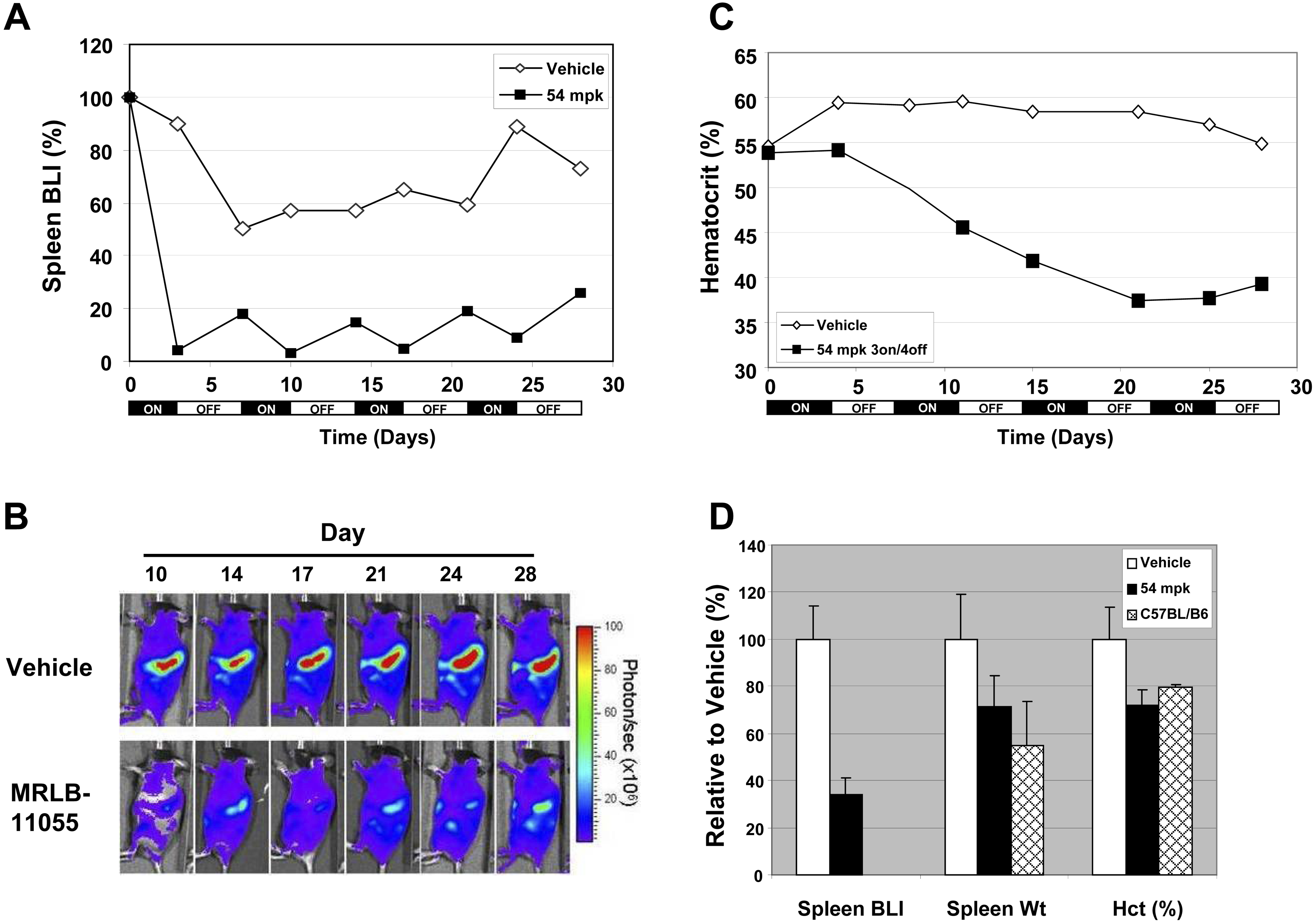

Supplement: Figure S2 — Effect of 4 Cycles of Intermittent Dosing (3 days on, 4 days off) of MRLB-11055 on V617F-Luc2 Mice (N = 10). Effect on A & B. Bioluminescence in spleen C. Hematocrit D. Multiple endpoints at end of study (Day 28). (TIF) [file pone.0037207.s002.tif]
