# Supplementary material for: Efficacious Intermittent Dosing of a Novel JAK2 Inhibitor in Mouse Models of Polycythemia Vera
Source: PLoS One. 2012 May 18;7(5):e37207. doi: 10.1371/journal.pone.0037207 (PMC3356383; doi:10.1371/journal.pone.0037207)
Supplement: Table S1 — Recombinant Kinase Selectivity Profile of MRLB-11055. IC50s measured at ATP Km, and fold increase relative to JAK2 is reported. (DOC) [file pone.0037207.s003.doc]

Table S1. Recombinant

Kinase Selectivity Profile of MRLB-11055

| **Kinase** | **Fold-Shift**  **/JAK2** |
| --- | --- |
| cSRC | 1 |
| TrkA | 1 |
| ACK1 | 2 |
| Fms | 2 |
| Fyn | 2 |
| Flt3 | 3 |
| Hck | 3 |
| Ret | 3 |
| Bmx | 4 |
| Lyn | 4 |
| Fgr | 5 |
| Lck | 5 |
| ARK5 | 6 |
| BTK | 7 |
| Yes | 7 |
| PTK5 | 8 |
| TrkB | 8 |
| CaMKIIδ | 10 |
| CaMKIIγ | 11 |
| PRK2 | 13 |
| Arg | 17 |
| MLK1 | 18 |
