# Supplementary material for: Efficacious Intermittent Dosing of a Novel JAK2 Inhibitor in Mouse Models of Polycythemia Vera
Source: PLoS One. 2012 May 18;7(5):e37207. doi: 10.1371/journal.pone.0037207 (PMC3356383; doi:10.1371/journal.pone.0037207)
Supplement: Table S2 — Effect of MRLB-11055 on Cell Populations in Spleen of Normal Mice. Cell counts measured by Advia. Cycles refer to 3 days of treatment followed by a 4 day holiday. *p<0.05 in Student T test when comparing vehicle 5 cycle with treatment 5 cycle and vehicle 2 cycle with treatment 2 cycle, resp. (DOC) [file pone.0037207.s004.doc]

Table S2. Effect of MRLB-11055 on Cell Populations in Spleen of Normal Mice

|  |  |  |  | B-Cells | | T-Cells | | | NK-Cells | Myeloid |
| --- | --- | --- | --- | --- | --- | --- | --- | --- | --- | --- |
| Group | n | Weight (mg) | Cell Count (X10^6^) | B220+ (X10^6^) | MZ-B-cells  CD21^hi^CD23^lo^  (X10^6^) | CD4+  (X10^6^) | Tregs  CD25+CD127^lo^  (X10^6^) | CD8+  (X10^6^) | NK1.1+ NKG2D+  (X10^6^) | Gr-1+ CD11b+  (X10^6^) |
| Vehicle  (2 cycles) | 6 | 83.2 ± 8.2 | 56.6 ± 5.8 | 33.9 ± 4.4 | ND | 9.6 ± 1.0 | 0.9 ± 0.1 | 5.9 ± 0.4 | 1.6 ±  0.2 | 0.57 ± 0.08 |
| 54 mpk  (2 cycles) | 3 | 76.3 ± 4.7 | 53.2 ± 1.2 | 30.9 ± 2.2 | ND | 6.5 ± 1.0* | 0.4 ± 0.1 * | 4.0 ± 0.4* | 1.2 ±  0.2* | 0.67 ± 0.17 |
|  |  |  |  |  |  |  |  |  |  |  |
| Vehicle  (5 cycles) | 4 | 82.3 ± 5.7 | 77.6 ± 16.6 | 42.3 ± 9.0 | 3.4 ± 0.4 | 14.3 ± 3.4 | 1.4 ± 0.3 | 10.4 ± 2.5 | 1.7 ±  0.5 | 0.55 ± 0.11 |
| 54 mpk  (5 cycles) | 4 | 92.5 ± 29.8 | 59.4 ± 20.3 | 31.2 ± 6.9 | 1.7 ± 0.4* | 7.9 ± 1.9* | 0.6 ± 0.2* | 4.9 ± 1.1* | 0.9 ±  0.3* | 1.05 ± 0.76 |
|  |  |  |  |  |  |  |  |  |  |  |
| 54 mpk  (3 days) | 4 | 50.5 ± 4.4* | 36.2 ± 8.7* | 24.2 ± 6.2* | 0.2 ± 0.2* | 4.9 ± 0.8* | 0.3 ± 0.1* | 4.7 ± 1.1* | 0.10 ± 0.03* | 0.25 ± 0.13* |
| 54 mpk  (6 days) | 4 | 45.8 ± 7.6* | 26.5 ± 3.1* | 16.9 ± 8.7* | 0.2 ± 0.2* | 4.1 ± 1.7* | 0.2 ± 0.1* | 3.2 ± 1.5* | 0.07 ± 0.04* | 0.22 ± 0.09* |
