# Supplementary material for: Efficacious Intermittent Dosing of a Novel JAK2 Inhibitor in Mouse Models of Polycythemia Vera
Source: PLoS One. 2012 May 18;7(5):e37207. doi: 10.1371/journal.pone.0037207 (PMC3356383; doi:10.1371/journal.pone.0037207)
Supplement: Table S3 — Effect of MRLB-11055 on Cell Populations in Peripheral Blood of Normal Mice. Cell counts measured by Advia. Cycles refer to 3 days of treatment followed by a 4 day holiday. *p<0.01 and **p<0.001 in Student T test when comparing vehicle with treatment. HCT, hematocrit; RBC, red blood cells; HGB, hemoglobin; WBC, white blood cells. (DOC) [file pone.0037207.s005.doc]

Table S3. Effect of MRLB-11055 on Cell Populations in Peripheral Blood of Normal Mice.

| Group | n | HCT  (%) | RBC  (x 10^6^/μl) | HGB  (g/dL) | Retic  (x 10^9^/L) | Platelets | | WBC  (x 10^3^/μl) | Lympho-cytes  (x 10^3^/μl) | Neutro-phils  (x 10^3^/μl) | Mono-cytes  (x 10^3^/μl) | Eosino-phils  (x 10^3^/μl) | Baso-phils  (x 10^3^/μl) |
| --- | --- | --- | --- | --- | --- | --- | --- | --- | --- | --- | --- | --- | --- |
|  |  |  |  |  |  | MPV fL | (x 10^3^/μl) |  |  |  |  |  |  |
| Vehicle  Controls | 12 | 54.3 ± 1.7 | 10.15 ± 0.29 | 14.8 ± 0.4 | 287 ±  37 | 6.5 ±  0.1 | 1333 ± 125 | 9.9 ±  2.0 | 8.5 ±  1.6 | 0.75 ± 0.17 | 0.06 ± 0.02 | 0.27 ± 0.15 | 0.05 ± 0.01 |
| 54 mpk  (2 cycles) | 5 | 51.6 ± 1.5* | 9.65 ± 0.27* | 13.4 ± 0.4** | 803 ±  18** | 6.9 ±  0.2** | 2244 ± 85** | 7.4 ±  1.7 | 6.2 ±  1.6 | 0.78 ± 0.18 | 0.04 ± 0.01 | 0.14 ± 0.06 | 0.03 ± 0.01 * |
| 54 mpk  (5 cycles) | 5 | 50.3 ± 0.6** | 9.41 ± 0.13** | 13.0 ± 0.1** | 911 ±  64** | 6.8 ±  0.1** | 2376 ± 171 ** | 5.9 ±  1.9* | 4.7 ±  1.5** | 0.86 ± 0.34 | 0.05 ± 0.01 | 0.06 ± 0.03 * | 0.04 ± 0.01 |
| 54 mpk  (3 days) | 5 | 50.4 ± 0.8** | 9.29 ± 0.04** | 13.7 ± 0.2** | 43 ±  6** | 6.6 ±  0.4 | 1611 ± 85** | 5.3 ±  1.0** | 4.2 ±  0.9** | 0.61 ± 0.07 | 0.05 ± 0.02 | 0.26 ± 0.09 | 0.03 ± 0.03 |
| 54 mpk  (6 days) | 5 | 48.7 ± 2.5** | 8.80 ± 0.39** | 12.9 ± 0.5** | 162 ±  24** | 6.1 ±  0.2** | 2534 ± 88** | 4.6 ±  0.6** | 3.5 ±  0.4** | 0.60 ± 0.16 | 0.05 ± 0.02 | 0.19 ± 0.06 | 0.02 ± 0.01** |
