# Supplementary material for: Efficacious Intermittent Dosing of a Novel JAK2 Inhibitor in Mouse Models of Polycythemia Vera
Source: PLoS One. 2012 May 18;7(5):e37207. doi: 10.1371/journal.pone.0037207 (PMC3356383; doi:10.1371/journal.pone.0037207)
Supplement: Table S4 — Effect of MRLB-11055 on Cell Populations in Bone Marrow of Normal Mice. Cell counts measured by Advia. Cycles refer to 3 days of treatment followed by a 4 day holiday. *p<0.05 in Student T test when comparing vehicle 5 cycle with treatment 5 cycle and vehicle 2 cycle with treatment 2 cycle, resp. (DOC) [file pone.0037207.s006.doc]

Table S4. Effect of MRLB-11055 on Cell Populations in Bone Marrow of Normal Mice.

| Group | n | Mature Erythrocytes  (x 10^6^) | Erythroid Progenitors  (x 10^6^) | Nucleated Cells  (x 10^6^) | Myeloid  GR-1+ CD11b+  (x 10^6^) | B-Cells (CD19+B220+) | | | |
| --- | --- | --- | --- | --- | --- | --- | --- | --- | --- |
|  |  |  |  |  |  | Total  (x 10^6^) | Pro/pre AA4.1+ IgM-  (x 10^6^) | Immature AA4.1+ IgM+  (x 10^6^) | Mature AA4.1- IgM+  (x 10^6^) |
| Vehicle (2 cycles) | 6 | 17.7 ± 4.9 | 6.9 ± 1.1 | 23.0 ± 2.9 | 6.24 ± 1.19 | 5.88 ± 0.89 | 3.54 ± 0.64 | 1.56 ± 0.22 | 0.73 ± 0.17 |
| 54 mpk (2 cycles) | 3 | 13.5 ± 0.7 | 8.3 ± 0.9 | 25.0 ± 4.0 | 8.33 ± 0.85* | 4.05 ± 0.94* | 2.78 ± 0.92 | 0.70 ± 0.04* | 0.54 ± 0.12 |
|  |  |  |  |  |  |  |  |  |  |
| Vehicle (5 cycles) | 4 | 14.1 ± 2.2 | 5.4 ± 0.3 | 18.6 ± 1.8 | 5.75 ± 0.56 | 3.81 ± 0.68 | 2.07 ± 0.38 | 0.74 ± 0.15 | 0.98 ± 0.21 |
| 54 mpk (5 cycles) | 4 | 10.3 ± 3.2 | 5.9 ± 1.9 | 19.6 ± 7.2 | 7.83 ± 3.46 | 1.76 ± 0.87* | 1.22 ± 0.74 | 0.15 ± 0.05* | 0.38 ± 0.17* |
|  |  |  |  |  |  |  |  |  |  |
| 54 mpk (3 days) | 4 | 25.0 ± 5.5* | 0.5 ± 0.3* | 4.7 ± 1.6* | 2.07 ± 0.55* | 0.78 ± 0.35* | 0.24 ± 0.10* | 0.22 ± 0.09* | 0.32 ± 0.17* |
| 54 mpk (6 days) | 4 | 18.7 ± 2.5* | 1.4 ± 0.8* | 5.4 ± 2.2* | 1.90 ± 0.68* | 0.79 ± 0.54* | 0.08 ± 0.03* | 0.11 ± 0.03* | 0.59 ± 0.52 |
