# Supplementary material for: Efficacious Intermittent Dosing of a Novel JAK2 Inhibitor in Mouse Models of Polycythemia Vera
Source: PLoS One. 2012 May 18;7(5):e37207. doi: 10.1371/journal.pone.0037207 (PMC3356383; doi:10.1371/journal.pone.0037207)
Supplement: Table S5 — Effect of MRLB-11055 on Cell Populations in Thymus of Normal Mice. Cell counts measured by Advia. Cycles refer to 3 days of treatment followed by a 4 day holiday. *p<0.05 in Student T test when comparing vehicle 5 cycle with treatment 5 cycle and vehicle 2 cycle with treatment 2 cycle, resp. (DOC) [file pone.0037207.s007.doc]

Table S5. Effect of MRLB-11055 on Cell Populations in Thymus of Normal Mice

| Group | n | Cell Count  (x 10^6^) | CD4+CD8+  (%) | Single Positive | | CD4-CD8-  (%) |
| --- | --- | --- | --- | --- | --- | --- |
|  |  |  |  | CD4+  (%) | CD8+  (%) |  |
| Vehicle (2 cycles) | 6 | 93.8 ± 24.0 | 88.8 ± 1.2 | 5.6 ± 0.5 | 1.2 ± 0.2 | 3.1 ± 0.7 |
| 54 mpk (2 cycles) | 3 | 75.4 ± 26.5 | 91.3 ± 0.3 | 3.9 ± 0.8* | 0.55 ± 0.03* | 3.0 ± 0.8 |
|  |  |  |  |  |  |  |
| Vehicle (5 cycles) | 4 | 138.5 ± 24.1 | 89.5 ± 1.1 | 5.4 ± 0.8 | 2.1 ± 0.2 | 2.2 ± 0.3 |
| 54 mpk (5 cycles) | 4 | 130.3 ± 27.1 | 90.7 ± 1.5 | 5.0 ± 1.1 | 1.4 ± 0.2* | 2.1 ± 0.6 |
|  |  |  |  |  |  |  |
| 54 mpk (3 days) | 4 | 37.3 ± 13.4* | 85.6 ± 2.4* | 7.1 ± 1.3 | 2.7 ± 0.8 | 3.5 ± 0.6 |
| 54 mpk (6 days) | 4 | 37.3 ± 12.1* | 87.9 ± 5.0 | 5.8 ± 2.7 | 2.7 ± 1.1 | 2.4 ± 0.9 |
